# The Rab32/BLOC-3–dependent pathway mediates host defense against different pathogens in human macrophages

**DOI:** 10.1126/sciadv.abb1795

**Published:** 2021-01-15

**Authors:** Massimiliano Baldassarre, Virtu Solano-Collado, Arda Balci, Rosa A. Colamarino, Ivy M. Dambuza, Delyth M. Reid, Heather M. Wilson, Gordon D. Brown, Subhankar Mukhopadhyay, Gordon Dougan, Stefania Spanò

**Affiliations:** 1 Institute of Medical Sciences, University of Aberdeen, Foresterhill, Aberdeen AB252ZD, UK.; 2 MRC Centre for Medical Mycology, University of Exeter, Geoffrey Pope Building, Stocker Road, Exeter EX4 4QD, UK.; 3 MRC Centre for Transplantation, Peter Gorer Department of Immunobiology, School of Immunology and Microbial Sciences, King’s College London, Great Maze Pond, London, SE1 9RT, UK.; 4 Wellcome Trust Sanger Institute, Hinxton, Cambridge CB10 1SA, UK.

## Abstract

Macrophages provide a first line of defense against microorganisms, and while some mechanisms to kill pathogens such as the oxidative burst are well described, others are still undefined or unknown. Here, we report that the Rab32 guanosine triphosphatase and its guanine nucleotide exchange factor BLOC-3 (biogenesis of lysosome-related organelles complex–3) are central components of a trafficking pathway that controls both bacterial and fungal intracellular pathogens. This host-defense mechanism is active in both human and murine macrophages and is independent of well-known antimicrobial mechanisms such as the NADPH (reduced form of nicotinamide adenine dinucleotide phosphate)–dependent oxidative burst, production of nitric oxide, and antimicrobial peptides. To survive in human macrophages, *Salmonella* Typhi actively counteracts the Rab32/BLOC-3 pathway through its *Salmonella* pathogenicity island-1–encoded type III secretion system. These findings demonstrate that the Rab32/BLOC-3 pathway is a novel and universal host-defense pathway and protects mammalian species from various pathogens.

## Teaser

The small GTPase Rab32 and its exchange factor BLOC-3 complex control a new antimicrobial activity in mouse and human macrophages.

## INTRODUCTION

Cells of our innate immune system, e.g., macrophages, are involved in the first line of defense against microorganisms. After phagocytosis, macrophages can eliminate most of the microorganisms they encounter by directing them in intracellular compartments where conditions are not compatible with microorganism life. A key strategy used by macrophages to kill microbes is the production of reactive oxygen species (ROS) through activation of the NADPH oxidase complex that is assembled on cellular membranes in response to infection (*1*). Other mechanisms, such as the production of nitric oxide, or cathelicidin-related antimicrobial peptide (Cramp), can also mediate bacterial killing (*2*). Despite the presence of a number of potent antimicrobial mechanisms, some microorganisms have evolved to become effective intracellular pathogens by escaping clearance and killing mechanisms present in macrophages and other immune cell types. For example, *Salmonella enterica* harbors two type III secretion systems that are responsible for the delivery of a battery of effectors that allow *Salmonella* to actively invade host cells, including macrophages, and survive in a specialized intracellular compartment known as the *Salmonella*-containing vacuole (SCV) (*3*–*5*). *S. enterica* is a genetically diverse bacterial species that includes hundreds of different serovars that can cause human and important veterinary diseases. *S. enterica* serovar Typhi (*S*. Typhi) is a human-restricted serovar that causes typhoid fever, a disease that affects ≈22 million people every year (*6*). Unlike many *Salmonella* serovars that can infect a broad range of hosts, *S*. Typhi naturally only infects humans (*7*). For example, it cannot establish an oral infection in laboratory mice (*8*).

Previously, we have shown that the inability of *S*. Typhi to infect mice depends, at least in part, on the fact that this pathogen cannot target the Rab32 GTPase in mouse macrophages (*9*). This GTPase and its guanine nucleotide exchange factor BLOC-3 (biogenesis of lysosome-related organelles complex–3) are central components of a pathway that regulate membrane trafficking to lysosome-related organelles in several specialized cell types (*8*, *9*). The murine Rab32/BLOC-3 pathway is effectively neutralized by the murine-virulent *S. enterica* serovar Typhimurium (*S.* Typhimurium) through the delivery of two *Salmonella* Pathogenicity Island 2 (SPI-2) type III secretion effectors, GtgE and SopD2, that directly target Rab32 by acting as a protease and a GTPase-activating protein, respectively (*8*, *10*). *S.* Typhimurium mutants defective for both these effectors are virtually avirulent in wild-type mice but are able to infect mice that are either deficient for Rab32 or BLOC-3 (*10*).

## RESULTS

**Fig. 1:**
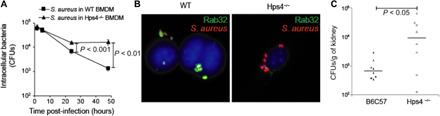
The Rab32/BLOC-3–dependent pathway mediates the killing of different pathogens. (**A** and **B**) BMDMs were derived from control mice C57BL/6 (wt) or from HPS4^−/−^ mice, infected with *S. aureus* and (A) CFUs were enumerated at the times indicated or (B) cells were fixed at 3 hours post-infection (p.i.) and stained to show Rab32 localization. (**C**) wt or HPS4^−/−^ mice were infected with *C. albicans*, and fungal burden in kidneys was evaluated 72 hours p.i.

Our previous work suggested that the Rab32/BLOC-3–dependent pathway limits the infectivity of bacteria that have not evolved to neutralize it. Therefore, we investigated whether this pathway can control other pathogens that are known to persist intracellularly. When bone marrow–derived macrophages (BMDMs) from wild-type, Rab32, or BLOC-3–deficient mice were infected with *Staphylococcus aureus*, we observed a substantial increase in intracellular persistence in BMDMs deficient for the Hermansky-Pudlak Syndrome 4 (HPS4) protein, one of the two subunits of BLOC-3, or Rab32 when compared to wild-type BMDMs (Fig. 1A and fig. S1). In line with this, Rab32 is recruited to the vacuole containing *S. aureus* in wild-type but not HPS4-deficient BMDMs (Fig. 1B). Given that *S. aureus* is a Gram-positive bacterium, these data suggested that Rab32 and BLOC-3 are components of an antimicrobial pathway that is important for the clearance of a number of different bacterial pathogens. Therefore, we investigated whether this pathway can also limit infection by fungal pathogens. Wild-type and HPS4-deficient mice were infected with *Candida albicans*, and the fungal burden in kidneys, the main organ affected by this pathogen, was evaluated 72 hours post-infection (p.i.). BLOC-3–deficient mice exhibited a 14-fold increase in kidney fungal colony-forming units (CFUs) (Fig. 1C). These results indicate that the Rab32/BLOC-3–dependent pathway is critical for defending the host from both bacterial and fungal attacks.

**Fig. 2:**
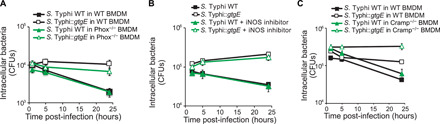
The Rab32/BLOC-3–dependent pathway does not require oxidative burst to clear bacterial and fungal infections in murine cells. BMDMs were infected with *S*. Typhi wild-type (WT) or expressing GtgE (*::gtgE*), and CFUs were enumerated at the times indicated. (**A**) BMDMs were derived from control mice (wt) or from NADPH oxidase^−/−^ mice (Phox^−/−^). (**B**) BMDMs were infected in the presence or absence of the iNOS inhibitor 1400W. (**C**) BMDMs were derived from control mice (wt) or CRAMP^−/−^ mice.

To investigate potential mechanisms for the Rab32-dependent clearance, we infected BMDMs from wild type and mice defective in particular antimicrobial factors with *S.* Typhi. ROS are molecules that are toxic to many species that have not evolved strategies to neutralize them (*11*). Innate immune cells can assemble phagocytic NADPH oxidase on the phagosome to generate ROS to kill intracellular pathogens (*12*). BMDMs derived from NADPH oxidase-deficient mice (Phox^−/−^) clear *S.* Typhi similarly to wild-type BMDM, while an *S.* Typhi strain engineered to deliver the protease GtgE that cleaves Rab32 (*8*) is not killed so efficiently in either macrophage (Fig. 2A). Similarly, the production of nitric oxide radicals by the inducible nitric oxide synthase (iNOS) and Cramp, two important mechanisms that control pathogenic species, is not essential to clear *S.* Typhi in murine BMDMs (Fig. 2, B and C). These data indicate that the Rab32/BLOC-3–dependent pathway works independently of these well-characterized mechanisms of pathogen clearance and that another unknown mechanism underpins that ability of the Rab32 pathway to clear *S.* Typhi infections in murine cells.

The broad-host *Salmonella* serovar *S.* Typhimurium delivers two type III secretion effectors GtgE and SopD2 that confer the ability of isolates of this serovar to infect mice (*8*, *9*, *13*). *S.* Typhi lacks these two effectors and cannot infect mice but is able to survive in human macrophages and cause a systemic infection in humans. These facts could suggest that the Rab32/BLOC-3–dependent host-defense pathway is not fully active in human macrophages. However, the Rab32 and BLOC-3 genes are present in humans and genome-wide association studies have shown that single-nucleotide polymorphisms in the Rab32 untranslated regions are associated with increased susceptibility to leprosy, a human bacterial infection caused by the intracellular bacterium *Mycobacterium leprae* (*14*, *15*). This suggests that Rab32 could be part of a pathway critical to control some bacterial infection in humans. Two scenarios could explain these findings: (i) Rab32/BLOC-3 are not part of a host-defense pathway in humans or (ii) a Rab32/BLOC-3–dependent pathway is active in humans as an antimicrobial mechanism, but *S*. Typhi has evolved molecular strategies to evade it.

**Fig. 3:**
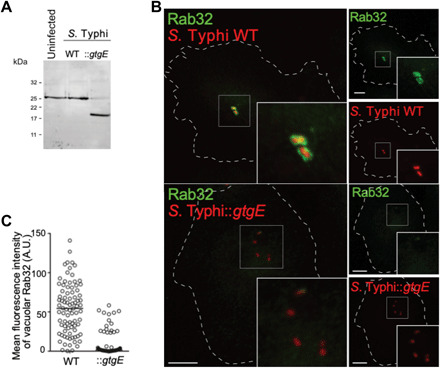
GtgE delivery from *S*. Typhi results in the cleavage of human Rab32. (**A**) PMA-differentiated THP-1 cells were left uninfected or infected with either wild-type *S*. Typhi (WT) or an *S*. Typhi strain expressing GtgE (::*gtgE*). Cells were lysed 2.5 hours p.i. and analyzed by Western blot with a Rab32-specific antibody. (**B** and **C**) Peripheral blood monocyte–derived macrophages were infected with either wild-type *S*. Typhi (WT) or an *S*. Typhi derivative expressing GtgE (::*gtgE*), both carrying a chromosomal copy of the *mCherry* gene, fixed at 2.5 hours p.i. and analyzed by immunofluorescence with a Rab32-specific antibody. Scale bars, 10 μm. A.U., arbitrary units.

To assess whether the Rab32/BLOC-3 host-defense pathway is active as an antimicrobial pathway in humans, we investigated the requirement of Rab32 and BLOC-3 in controlling bacterial growth in human macrophages. We used an *S*. Typhi strain engineered to express the *S.* Typhimurium type III secretion effector GtgE, a specific protease that cleaves the three Rab GTPases, Rab32, Rab29, and Rab38 (*8*, *16*). We infected human macrophage-like THP-1 cells with an *S*. Typhi wild-type isolate [ISP2825 (*17*)] or an isogenic strain engineered to express GtgE (*S*. Typhi::*gtgE*). GtgE delivery from *S*. Typhi results in the cleavage of human Rab32 (Fig. 3A), indicating that GtgE can target endogenous human Rab32, in agreement with the previous observation that GtgE cleaves ectopically expressed human Rab32 (*8*). When we infected human blood monocyte–derived primary macrophages, we observed that Rab32 localizes on the surface of the vacuoles containing wild-type *S*. Typhi but is mostly absent from the surface of the vacuoles containing *S*. Typhi::*gtgE* (Fig. 3, B and C).

**Fig. 4:**
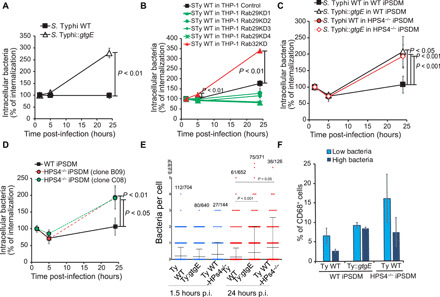
Rab32 inactivation and BLOC-3 knockout results in *S*. Typhi over-replication in human macrophages. (**A**) Peripheral blood monocyte–derived macrophages were infected with either wild-type *S*. Typhi (WT) or an *S*. Typhi strain expressing GtgE (::*gtgE*). Cells were lysed at the indicated time points to measure CFUs. (**B**) THP-1 cells were transduced with lentivirus to silence the indicated Rab, infected with wild-type *S*. Typhi, and lysed at the indicated time points to measure CFUs. (**C**) Human macrophages derived from WT or HPS4^−/−^ hiPSCs were infected with either wild-type *S*. Typhi (WT) or an *S*. Typhi strain expressing GtgE (::*gtgE*), lysed at the indicated time points for counting of intracellular CFUs. (**D**) Human macrophages derived from WT or HPS4^−/−^ hiPSCs were infected with wild-type *S*. Typhi (WT) and lysed at the indicated time points to measure CFUs. (**E**) Human macrophages derived from WT or HPS4^−/−^ hiPSCs were plated on glass coverslips, then infected with *S*. Typhi *glmS∷Cm::mCherry* or *S*. Typhi::*gtgE glmS∷Cm::mCherry*, and fixed at 1.5 and 24 hours p.i. Differentiated macrophages were identified by CD68 staining, and bacteria in CD68^+^ cells were counted. The whole populations are reported, and the infected versus total number of cells are indicated. Bars represent the mean and SD of the population. (**F**) Human macrophages derived from WT or HPS4^−/−^ hiPSCs were infected with *S*. Typhi *glmS∷Cm::mCherry* or *S*. Typhi::*gtgE glmS∷Cm::mCherry*, fixed at 24 hours p.i., and analyzed by flow cytometry. CD68^+^ cells were subgated in two populations containing respectively low or high mCherry signal (i.e., bacterial content). Errors bars represent SD between experiments.

We then investigated whether the removal of Rab32 from the bacterial vacuole has any effect on *S*. Typhi survival. GtgE expression confers *S*. Typhi a threefold replicative advantage in blood monocyte–derived primary macrophages at 24 hours p.i. (Fig. 4A), suggesting that one of the three Rab GTPases targeted by GtgE (*8*, *16*) controls *S*. Typhi intracellular survival in human macrophages. As Rab38 mRNA is hardly detectable in either THP-1 or primary macrophages (fig. S2 and “The Human Protein Atlas”), we analyzed whether either Rab32 or Rab29 is responsible for the limitation of *S*. Typhi growth in human macrophages by knocking down either Rab29 or Rab32 from THP-1 cells (>70 and >80% knockdown, respectively; fig. S2). While depletion of Rab32 resulted in a significantly increased replication of *S*. Typhi (Fig. 4B), only a slightly reduced replication was observed when Rab29 was depleted. Together, these results indicate that Rab32 is critical to control *S*. Typhi infections in human macrophages.

We then used macrophages derived from human-inducible pluripotent stem cells (hiPSCs), a recently established model for the study of *Salmonella* infection (*18*, *19*). First, we confirmed that, similarly to what was observed in THP-1 and primary macrophages, GtgE expression also confers an advantage to *S*. Typhi in hiPSC-derived macrophages (Fig. 4C). Next, we used CRISPR-Cas9 technology to generate hiPSCs deficient for HPS4. As shown in Fig. 4D, macrophages derived from two independent clones of HPS4-deficient hiPSCs have a significant increased number of *S*. Typhi intracellular CFUs at 24 hours p.i., demonstrating that BLOC-3 is important to limit *S*. Typhi growth in human macrophages. To obtain further insights into the effects of GtgE expression in *S*. Typhi or the knockout of BLOC-3 in these macrophages, we measured the number of *S*. Typhi in single cells by immunofluorescence (Fig. 4E) or flow cytometry analysis (Fig. 4F). These experiments confirmed that removal of either Rab32, obtained through GtgE delivery, or BLOC-3 results in an increased percentage of host cells containing higher numbers of bacteria. This indicates that both Rab32 and its guanine nucleotide exchange factor BLOC-3 are required for the control of *Salmonella* survival and replication in human macrophages. We also observed that although *S*. Typhi has a replicative advantage in the absence of BLOC-3, the expression of GtgE does not confer any significant additional advantage (Fig. 4C), in agreement with the model that Rab32 and BLOC-3 are components of the same pathway. The results of these experiments indicate that the Rab32/BLOC-3–dependent pathway is active as a host-defense pathway in human macrophages and can limit *S*. Typhi replication.

**Fig. 5:**
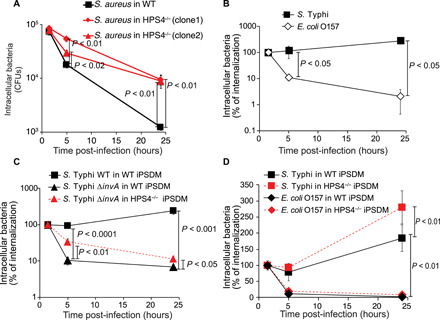
*S*. Typhi counteracts the Rab32/BLOC-3–dependent pathway through the SPI-1 type III secretion system. (**A**) Human macrophages derived from WT or HPS4^−/−^ hiPSCs were infected with *S. aureus* and lysed at the indicated time points to measure intracellular CFUs. (**B**) Human macrophages derived from WT hiPSCs were infected with wild-type *S*. Typhi (WT) or *E. coli* O157 and lysed at the indicated time points to measure intracellular CFUs. (**C**) Human macrophages derived from WT or HPS4^−/−^ hiPSCs were infected with either wild-type *S*. Typhi (WT) or *S*. Typhi ∆*invA* (∆*invA*). Cells were lysed at the indicated time points to measure intracellular CFUs. (**D**) Human macrophages derived from WT or HPS4^−/−^ hiPSCs were infected with either wild-type *S*. Typhi (WT) or *E. coli* O157. Cells were lysed at the indicated time points to measure intracellular CFUs. Results in (B), (C), and (D) are reported as percentage of CFUs measured at the first time point (1.5 hours p.i.). Values are means ± SEM of at least three independent experiments performed in triplicate. *P* values were calculated using the Student’s *t* test and are indicated only when <0.05.

To test whether the human Rab32/BLOC-3–dependent pathway exerts broad antimicrobial activity, we infected macrophages derived from two independent clones of HPS4-deficient hiPSCs with *S. aureus*. As shown in Fig. 5A, HPS4 knockout results in ≈10-fold increased survival of *S. aureus* in human macrophages. However, in contrast to *S. aureus* (Fig. 5A) and other pathogens, such as *Escherichia coli* O157 (Fig. 5B), *S*. Typhi is not as efficiently cleared by wild-type human macrophages during infection but instead persist in the majority of infected cells (Figs. 5B and 4, A to D). Therefore, we hypothesized that *S*. Typhi actively counteracts the pathway controlled by Rab32/BLOC-3. Because the broad-host *S.* Typhimurium neutralizes this pathway through the action of effectors delivered by type III secretion systems, we tested whether *S*. Typhi survival in wild-type human macrophages is dependent on *S*. Typhi type III secretion systems. We observed that *S*. Typhi survival in hiPSC macrophages is dependent on its SPI-1 (Fig. 5C), but not on its SPI-2 type III secretion system (fig. S3). An SPI-1 type III secretion system mutant of *S*. Typhi (*S*. Typhi∆*invA*) was unable to survive in macrophages derived from hiPSCs (Fig. 5C), in agreement with published results (*20*). *S*. Typhi∆*invA* survived much better in HPS4-deficient macrophages (Fig. 5C), suggesting that the Rab32/BLOC-3 pathway is involved in *S*. Typhi killing and that *S*. Typhi needs this secretion system to counteract this pathway. To confirm that HPS4 removal did not result in a completely impaired bacterial killing, we infected HPS4-deficient macrophages with pathogenic *E. coli* O157. This pathogen is not able to survive in either wild-type or HPS4-deficient macrophages (Fig. 5D). These results indicate that *S*. Typhi is able to target the human Rab32/BLOC-3–dependent pathway likely through expression of the SPI-1 type III secretion system. As *S*. Typhi cannot neutralize the mouse Rab32/BLOC-3–dependent pathway, we suggest that *S*. Typhi targets a human-specific component of this pathway.

## DISCUSSION

Overall, we show that Rab32 and BLOC-3 regulate host-defense activity against a variety of pathogens, including Gram-negative and Gram-positive bacterial pathogens and the fungal pathogen *C. albicans*. This activity is critical for the clearance of *S. aureus* and *S*. Typhi in mouse macrophages and for the clearance of *S. aureus* in human macrophages but is dispensable for *E. coli* killing. While neither the overall antimicrobial mechanism nor mechanisms controlled by Rab32 are yet defined, we show here that they do not require the production of ROS by the phagocytic NADPH oxidase, an ancient and broad antimicrobial mechanism active in macrophages. We also demonstrate that this activity does not require production of nitric oxide by iNOS and the macrophage antimicrobial peptide CRAMP.

In agreement with previous studies (*21*), we found that removal of Rab32 induces a general decrease in lysosome acidification in human macrophages (fig. S4). However, considering that *Salmonella* is resistant to the low phagosome pH in macrophages (*22*), it is not clear how this decrease in acidification can result in a substantial increase in intracellular persistence. It is also possible that Rab32 and BLOC-3 control different antimicrobial mechanisms; however, while this paper was under revision, a new study by Chen *et al.* (*23*) has shown that Rab32 is important for the delivery of itaconic acid to the SCV resulting in bacterial killing.

In contrast to *S. aureus*, *S*. Typhi uses its SPI-1 type III secretion system to evade this pathway in humans and persist in human macrophages. Not dissimilar from *S*. Typhimurium, which has evolved to deliver the protease GtgE and the Rab GAP SopD2 that act redundantly to inactivate the murine Rab32/BLOC-3 trafficking pathway, *S*. Typhi appears to have evolved a strategy to target the human Rab32/BLOC-3–dependent pathway that requires its SPI-1 type III secretion system. A possible reason for evolving a different strategy could rely on the fact that GtgE also targets Rab29, which is required for the efficient delivery of typhoid toxin from *S*. Typhi–infected cells (*16*). Therefore, we speculate that GtgE, although able to cleave the human Rab32, would not confer, overall, an advantage to *S*. Typhi because it would interfere with other pathogenic features of this bacterium, including the delivery of typhoid toxin. Similar to *S*. Typhimurium, *S*. Typhi may have evolved a number of redundant effectors with different activities to target the Rab32/BLOC-3-dependent pathway. In conclusion, here, we reveal that Rab32 and BLOC-3 control a novel antimicrobial activity against a variety of infectious pathogens and demonstrate that *S*. Typhi uses its SPI-1 type III secretion system to counteract this pathway and survive in human macrophages.

## MATERIALS AND METHODS

### Bacterial strains and plasmids

The wild-type *S* Typhi strain ISP2825 has been previously described (*17*). All the *S*. Typhi deletion strains were constructed by standard recombinant DNA and allelic exchange procedures as previously described (*24*) and are listed in table S1. All the plasmids used in this study were constructed using standard recombinant DNA techniques and are listed in table S2. *S*. Typhi *glmS∷Cm::mCherry* and *S*. Typhi::*gtgE glmS::Cm::mCherry* that constitutively express *mCherry* from a single chromosomal copy at the *att*Tn7 site were generated by P22 transduction using phages obtained from the *S*. Typhimurium SL1344 *glmS::Cm::mCherry* [a gift from L. Knodler; (*25*)]. HPS4^−/−^ (strain B6.C3-Pde6brd1 Hps4le/J) was purchased from the Jackson Laboratory.

### Cell culture

THP-1 cells were maintained in RPMI 1640 medium (Invitrogen), 10% fetal bovine serum (FBS; Invitrogen), 2 mM glutamine (Invitrogen), 1 mM sodium pyruvate (Invitrogen), and 10 mM Hepes (Invitrogen). The cells were maintained at a concentration between 0.1 and 1 million cells/ml. THP-1 differentiation was induced by adding 100 nM phorbol 12-myristate 13-acetate (PMA) for 48 hours before infection. For intracellular growth experiments, THP-1 differentiated cells were treated with human interferon-γ (IFN-γ) (150 ng/ml) 24 hours before infection. Human embryonic kidney 293T cells were maintained in Dulbecco’s modified Eagle’s medium (DMEM) high glucose, 2 mM Glutamax (Invitrogen), and 10% FBS.

Blood was collected from healthy human volunteers, according to procedures approved by the Life Science and Medicine College Ethics Review Board of the University of Aberdeen (CERB/2016/11/1299). Peripheral blood monocyte–derived macrophages were prepared as described in (*26*) with some modifications. Briefly, 13 ml of blood was collected from each donor, diluted to 35 ml of Hanks’ balanced salt solution (HBSS; Invitrogen), and then loaded onto 15 ml of Lymphoprep (Stem Cell Technology) for the separation of the peripheral blood mononuclear cells. Isolated peripheral blood mononuclear cells were resuspended in DMEM containing 10% autologous human serum (freshly prepared from the same donor) and seeded on coverslips or tissue-treated plastic. Cells were plated at 5 ×10^5^ per well in 24-well plates. After 24 hours, the nonadherent cells were removed, fresh medium was added, and the cells were left for 7 to 9 days to differentiate.

Undifferentiated human-induced pluripotent stem cells line (KOLF2-C1) was maintained on a monolayer of mitotically inactivated mouse embryonic feeder cells in advanced DMEM/F12 medium, supplemented with 20% knockout replacement serum (Invitrogen), 2 mM l-glutamine, 0.055 mM β-mercaptoethanol (Sigma-Aldrich), and recombinant human fibroblast growth factor 2 (FGF2) (8 ng/ml; R&D Systems), as described previously (*18*). These cells were differentiated into macrophages as described in a previously published method (*18*).

### *C. albicans* infection in wild-type or HPS4^−/−^ mice

*C. albicans* (strain SC5314) was serially grown overnight at 30°C with shaking. Yeast cells were washed in phosphate-buffered saline (PBS; Sigma-Aldrich), counted, and injected intravenously via the lateral tail vein. Animals were infected with 2 × 10^5^ CFUs. For analysis of fungal burdens in the kidneys, animals were euthanized 72 hours p.i. Kidneys were weighed, homogenized in PBS, and serially diluted before plating on to YPD (yeast extract, peptone, and dextrose) agar supplemented with penicillin/streptomycin (Invitrogen). Colonies were counted after incubation at 37°C for 24 to 48 hours.

### CRISPR-Cas9 targeting of HPS4

Isogenic intermediate targeting vectors for HPS4 were generated using isogenic and haplotype-specific DNA by polymerase chain reaction (PCR) amplification of KOLF2-C1 genomic DNA (gDNA). First, a PCR fragment including homology arms and the critical exon of HPS4 was amplified from KOLF2-C1 gDNA using the following primers: f5F gccagtgaattcgatatacctgccttcttgaactgttttg and f3R tacgccaagcttgatttaaattgtgctctgtgtgttcctc. The first 15 nucleotides (nt) of each primer (underlined) served to mediate fusion with the intermediate targeting vector backbone, puc19_RV, using an In-Fusion HD Cloning Plus kit (Takara Bio). The HSP4 amplicon was purified, and 75 ng was incubated with 50 ng of Eco RV–digested puc19_RV vector for 15 min at 50°C and transformed into Stellar competent cells (Takara Bio). Positive clones were verified by Sanger sequencing. To replace the critical exon with the gateway R1-*pheS/zeo*-R2 cassette, sequence-verified clones were electroporated with the pBABgbaA plasmid (*27*). This was then maintained in tetracycline (5 μg/ml) at 30°C. Early log-phase cultures were induced to express the red operon following addition of 0.1% arabinose and incubation for 40 min at 37°C. From these cultures, electrocompetent cells were prepared as previously described (*25*). The R1-*pheS/zeo*-R2 cassette was amplified using the following primers: U5 ttagtggtgtcagcagttctgagtatagagaggtagaatagtcccaagccaaggcgcataacgataccac and D3 agttgtgcagcaagggaatggggctggaagaaaggggtctggagttactcccgcctactgcgactataga. Underlined sequences in each of these primers denote 50 nt of homology toward a region 5′ (U5) or 3′ (D3) of the critical exon. This amplicon was purified, and 300 ng was electroporated into the recombination ready verified clones from the first step before selection in carbenicillin (50 μg/ml) and zeocin (10 μg/ml). Positive clones were verified by Sanger sequencing. To generate the donor plasmid for precise gene targeting via homology-directed repair, the intermediate targeting vectors were turned into donor plasmids via a Gateway exchange reaction. LR Clonase II Plus enzyme mix (Invitrogen) was used to perform a two-way reaction exchanging only the R1-*pheS/zeo*-R2 cassette with the pL1-EF1αPuro-L2 cassette as previously described (*27*). The latter had been generated by cloning synthetic DNA fragments of the EF1α promoter and puromycin resistance cassette into a pL1/L2 vector (*27*).

As part of the primer design process, two separate guide RNAs (gRNAs) targeting within the same critical exon were selected. The gRNAs were identified using the Wellcome Sanger Institute Genome Editing CRISPR tool (*28*) and were selected on the basis of their off-target scores to minimize potential off-target damage. gRNAs were suitably positioned to ensure DNA cleavage within the exonic region, excluding any sequence within the homology arms of the targeting vector. Plasmids carrying single gRNA sequences were generated by cloning forward and reverse strand oligos into the Bsa I site of either U6_Bsa I_gRNA or p1260_T7_Bsa I_gRNA vectors (provided by S. Gerety). The CRISPR sequences are as follows [protospacer adjacent motif (PAM) sequence]: left CRISPR (CCA), GCGAGAATGTGAGGGCGAGCG; and right CRISPR (CCT), TCAGCAACAACAGGGGCTCC (WGE IDs: 1181940311 and 1181940319, respectively).

To deliver plasmids expressing gRNA, donor templates, and Cas9, 2 × 10^6^ KOLF2-C1 cells were nucleofected (AMAXA nucleofector 2B) with 2 μg of donor plasmid, 4 μg of hCas9 D10A (Addgene, plasmid #41816) (*29*), and 3 μg of gRNA plasmid DNA. Following nucleofection, cells were selected for up to 11 days with 0.25 μg ml^−1^ puromycin. Individual colonies were picked into 96-well plates, expanded, and genotyped. Positive insertion of the cassette into the correct locus was confirmed by PCR using cassette-specific primers ER (gcgatctctgggttctacgttagtg) and PNFLR (catgtctggatccgggggtaccgcgtcgag). To determine the presence of deleterious insertions or deletions (indels) around the CRISPR target site of the opposite allele, a PCR amplicon was generated using the primers PR (actagttctaacagctggtggatac) and PF (ttttgcagactgacaactattccag), purified, and Sanger-sequenced using SR1 (cttctggacaggcctccttg) and SF1 (atatttgccgaaccagccca). To minimize the potential for off-target effects, two independently derived clones, B09 and C08, with specific deletions of 47 and 29 base pairs, respectively, were isolated and used in this study.

### Intracellular growth experiments

Overnight cultures of the different *S.* Typhi strains or *S. aureus* [strain SH1000 (*30*)] were diluted 1/20 in LB broth containing 0.3 M NaCl and grown for 2 hours and 45 min at 37°C. Cells were infected with the different strains of *S*. Typhi in HBSS at the desired multiplicity of infections. One-hour p.i. cells were washed three times with HBSS and incubated in growth medium supplemented with gentamicin (100 μg/ml) for 30 min to kill extracellular bacteria. Cells were then washed with HBSS, and fresh DMEM containing gentamicin (5 μg/ml) was added to avoid cycles of reinfection. At the indicated time points, the cells were washed twice in PBS, and the intracellular bacteria recovered lysing the cells in 0.1% sodium deoxycholate (*S. enterica*) or 0.1% Triton X-100 (Sigma-Aldrich) (*S. aureus*) in PBS were counted by plating serial dilutions on LB-agar plates.

### Western blot

PMA-differentiated THP-1 cells were infected as described above and lysed in SDS–polyacrylamide gel electrophoresis loading buffer 2.5 hours p.i. Western blot analysis was performed using the Odyssey Infrared Imaging System (LI-COR Biosciences). The following antibodies were used for Western blot analysis: rabbit polyclonal anti-Rab32 (GeneTex; 1:1000 dilution) and donkey anti-rabbit IR Dye 800 (LI-COR Biosciences; 1:10,000 dilution).

### Rab32 and Rab29 knockdown in THP-1

THP-1 cells were transduced with lentivirus expressing short hairpin RNA targeting Rab29 (TRCN0000299449, TRCN0000303685, TRCN0000381042, and TRCN0000303621, Sigma-Aldrich) or Rab32 (TRCN0000047746, Sigma-Aldrich). Twenty-four hours after transduction, cells were treated with puromycin (5 μg/ml) to kill the nontransduced cells and kept in culture for not more than 2 weeks. Seventy-two hours before the infection, the cells were treated with 100 nM PMA for 48 hours to induce differentiation. Twenty-four hours before the infection, the PMA was removed, and the cells were stimulated with human IFN-γ (100 ng/ml) and then finally infected with *S*. Typhi as described above.

### Immunofluorescence

Bone marrow–derived mouse macrophages, human monocyte-derived macrophages, and wild-type or HPS4-deficient iPSDM were plated on glass coverslips (#1, Thermo Fisher Scientific) infected with different *S*. Typhi strains or with *S. aureus* [strain SH1000 (*30*)] and fixed at the indicated times p.i. with 4% paraformaldehyde (PFA) for 10 min. Cells were then permeabilized for 20 min by incubating in 0.02% saponin (Sigma-Aldrich), 0.2% BSA (Sigma-Aldrich), and 50 mM NH_4_Cl (Sigma-Aldrich) in PBS and incubated for 1 hour with monoclonal mouse anti-CD68 (KP1, Invitrogen; 1:200 dilution). Alternatively, cells were permeabilized for 20 min by incubating in 0.2% Triton X-100 (Sigma-Aldrich), 0.2% BSA (Sigma-Aldrich), and 50 mM NH_4_Cl (Sigma-Aldrich) in PBS and incubated for 1 hour with a rabbit polyclonal anti-Rab32 (GeneTex; 1:200 dilution). Cells were then stained using the appropriate Alexa Fluor 488– or Alexa Fluor 555–conjugated secondary antibodies (Invitrogen). Images were acquired using either a Nikon (Eclypse Ti2) equipped with a CFI (chromatic aberration free infinity) Plan Apochromat 100× objective and a Prime 95B 25-mm complementary metal oxide semiconductor (CMOS) camera (Photometrics) or a PerkinElmer Spinning disk confocal equipped with an ORCA Flash 4.0 CMOS camera (Hamamatsu). Images were analyzed using the respective software (Nikon Elements or Volocity).

### Live-cell fluorescence microscopy

Live-cell imaging experiments were performed at 37°C in a temperature-controlled chamber. Differentiated THP-1 cells plated on glass-bottom four-well chambers (1 × 10^5^ cells) were infected with *S*. Typhi *glmS∷Cm::mCherry* at a multiplicity of infection of 10. Five hours p.i., cells were stained with 1 μM LysoSensor Green DND-189 (Thermo Fisher Scientific) for 10 min, washed three times with PBS, and imaged using a Nikon fluorescence microscope (Eclypse Ti2) equipped with a CFI Plan Apochromat 100× objective and a Prime 95B 25-mm CMOS camera (Photometrics).

To quantify the intensity of fluorescence of LysoSensor Green DND-189 in the SCV, a macro was developed for the ImageJ software. Briefly, the *S*. Typhi *glmS∷Cm::mCherry* images were used to select the area of individual SCV and that selection was then used in the corresponding LysoSensor Green DND-189 image. The fluorescence value obtained for each SCV in both Rab32 knockdown and control cells was normalized on the basis of the maximal intensity value of control cells.

The level of cell fluorescence was quantified using ImageJ software, and the level of corrected total cell fluorescence (CTCF) was calculated as follows: CTCF = Integrated Density – (area of selected cell × mean fluorescence of background readings). The values obtained were normalized on the basis of the maximal intensity value of control cells.

### Flow cytometry

hiPSC-derived macrophages wild type or HPS4 deficient were plated on non–tissue culture–treated six-well plates (Thermo Fisher Scientific) and infected with *S*. Typhi *glmS::Cm::mCherry* or *S*. Typhi::*gtgE glmS::Cm::mCherry*. At the indicated time p.i., the cells were detached using 500 μl of Versene (Invitrogen) and mixed with an equal volume of 4% PFA for 5 min. Fixed cells were then centrifuged and resuspended in 4% PFA for 5 min. The cells were then transferred in flow cytometry tubes, permeabilized for 15 min in PMZ-S (50 mM NH_4_Cl, 0.5 % bovine serum albumin, 0.05% saponin), and then incubated for 1 hour with anti-CD68 (1:200) and then with anti-mouse Alexa Fluor 488. The samples were analyzed by flow cytometry (Fortessa, BD Biosciences) and FlowJo software.

### Statistical analysis

CFU data in macrophages are presented as mean ± SEM. Differences between two groups were analyzed using the appropriate paired or unpaired Student’s *t* test. For *C. albicans* CFUs (Fig. 1C), the indicated *P* values were determined by one-way analysis of variance (ANOVA) test with Dunnett’s posttest. *P* values of 0.05 or less were considered to be statistically significant. Excel (Microsoft) and GraphPad Prism7 (GraphPad Software Inc.) were used to perform all statistical analyses.

## Supplementary Material

http://advances.sciencemag.org/cgi/content/full/7/3/eabb1795/DC1

Adobe PDF - abb1795_SM.pdf

The Rab32/BLOC-3–dependent pathway mediates host defense against different pathogens in human macrophages

## SUPPLEMENTARY MATERIALS

Supplementary material for this article is available at http://advances.sciencemag.org/cgi/content/full/7/3/eabb1795/DC1

## Appendix

View/request a protocol for this paper from *Bio-protocol*.
