# Supplementary material for: The Rab32/BLOC-3–dependent pathway mediates host defense against different pathogens in human macrophages
Source: Sci Adv. 2021 Jan 15;7(3):eabb1795. doi: 10.1126/sciadv.abb1795 (PMC7810368; doi:10.1126/sciadv.abb1795)
Supplement: http://advances.sciencemag.org/cgi/content/full/7/3/eabb1795/DC1 [file supp_7_3_eabb1795__index.html]

Science Advances | Science AdvancesAAASSearchScience AdvancesMenu

## Supplementary Materials

# The Rab32/BLOC-3–dependent pathway mediates host defense against different pathogens in human macrophages

Massimiliano Baldassarre, Virtu Solano-Collado, Arda Balci, Rosa A. Colamarino, Ivy M. Dambuza, Delyth M. Reid, Heather M. Wilson, Gordon D. Brown, Subhankar Mukhopadhyay, Gordon Dougan, Stefania Spanò

Download Supplement

**This PDF file includes:**

- Figs. S1 to S4
- Tables S1 and S2

**Files in this Data Supplement:**

- Adobe PDF - abb1795\_SM.pdf
