# Supplementary material for: The Rab32/BLOC-3–dependent pathway mediates host defense against different pathogens in human macrophages
Source: Sci Adv. 2021 Jan 15;7(3):eabb1795. doi: 10.1126/sciadv.abb1795 (PMC7810368; doi:10.1126/sciadv.abb1795)
Supplement: http://advances.sciencemag.org/cgi/content/full/7/3/eabb1795/DC1 [file supp_7_3_eabb1795__1.pdf]

Science Advances
AAAS

advances.sciencemag.org/cgi/content/full/7/3/eabb1795/DC1

Supplementary Materials for

The Rab32/BLOC-3–dependent pathway mediates host defense against different pathogens in human macrophages

Massimiliano Baldassarre*, Virtu Solano-Collado, Arda Balci, Rosa A. Colamarino, Ivy M. Dambuza, Delyth M. Reid, Heather M. Wilson, Gordon D. Brown, Subhankar Mukhopadhyay, Gordon Dougan, Stefania Spanò

*Corresponding author. Email: massimiliano.baldassarre@abdn.ac.uk

Published 15 January 2021, *Sci. Adv.* **7**, eabb1795 (2021)
DOI: 10.1126/sciadv.abb1795

This PDF file includes:

Figs. S1 to S4
Tables S1 and S2

Fig S1

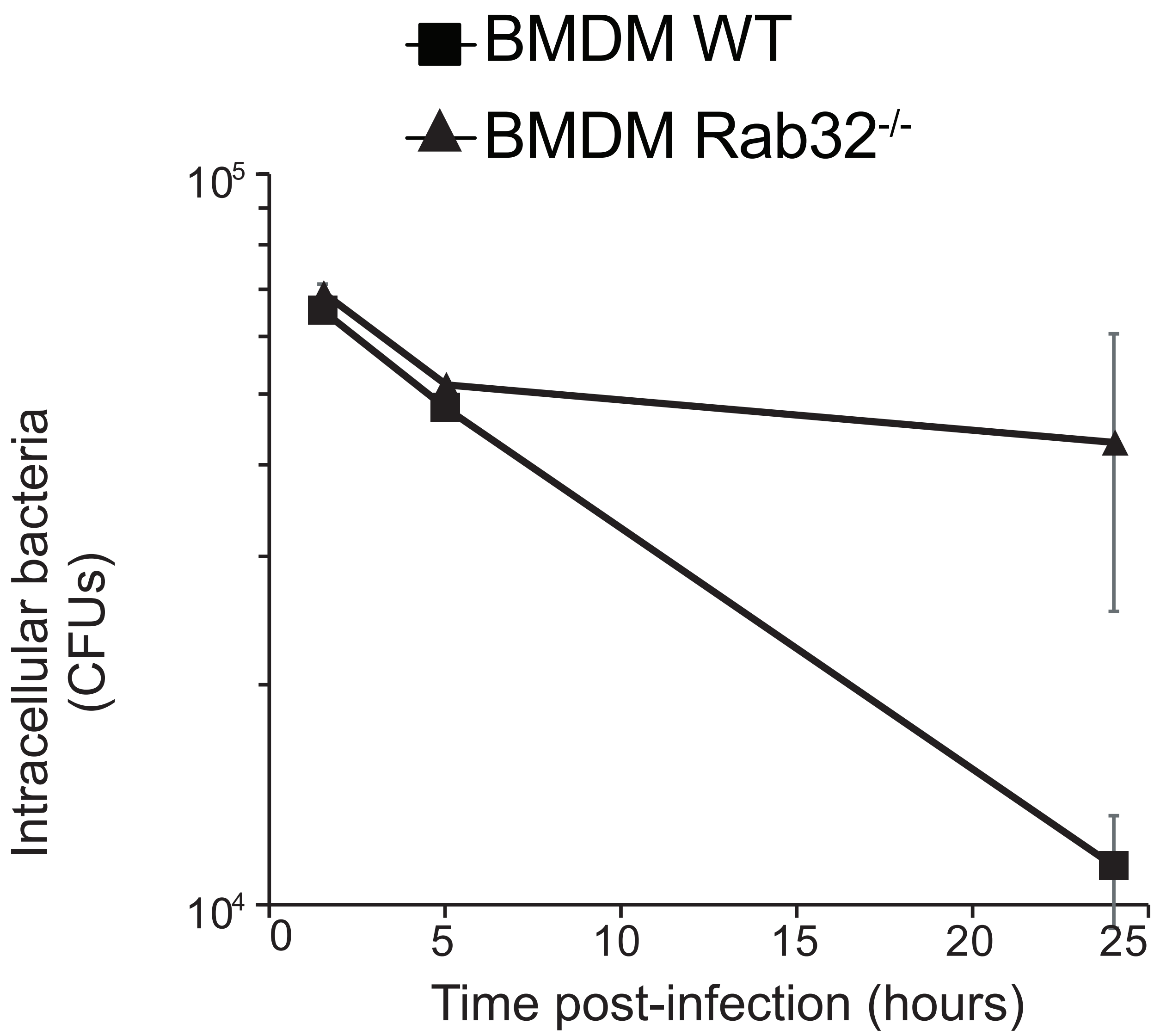

Fig S1. The Rab32/BLOC-3 pathway mediates the killing of different pathogens. Mouse bone marrow derived macrophages (BMDM) were derived from control mice C57BL/6 (wt) or from Rab32$^{-/-}$ mice were infected with *S. aureus*. Cells were lysed at the indicated time points to measure intracellular CFUs.

Fig S2

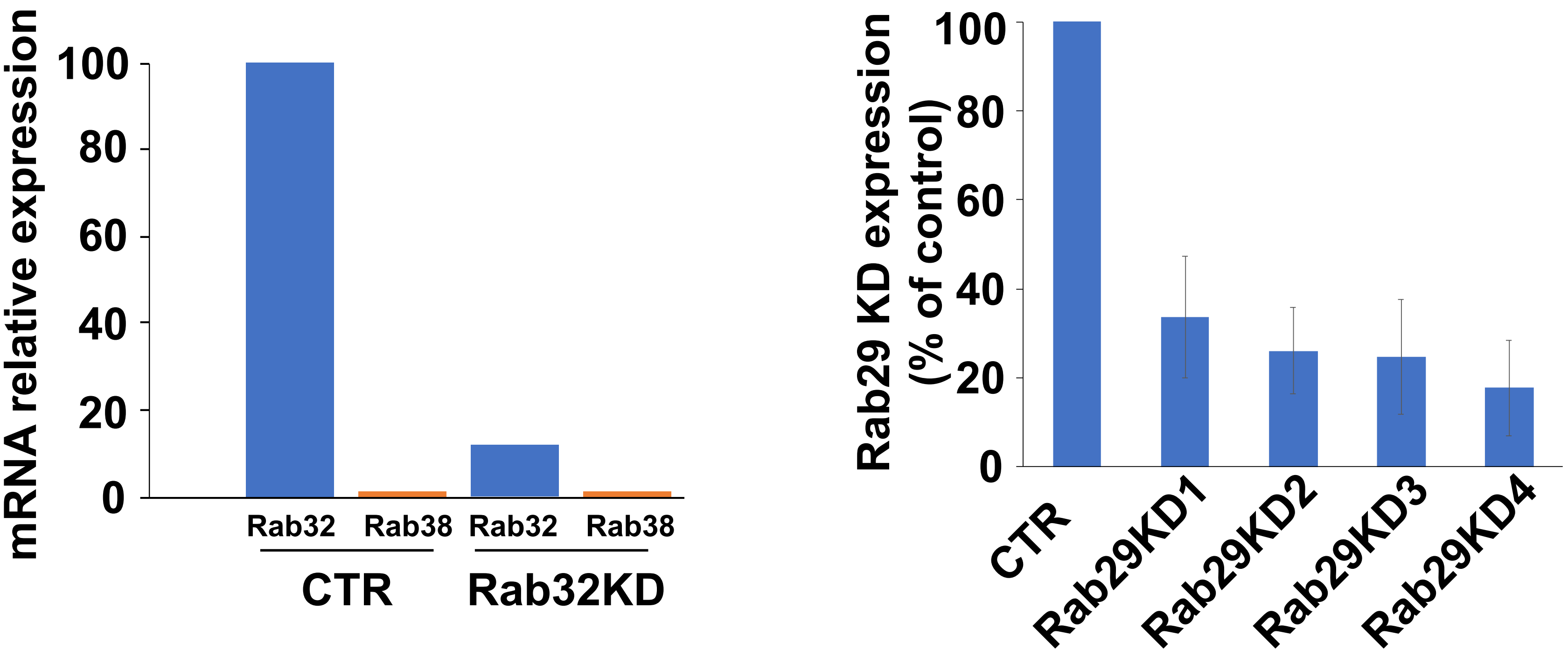

Figure S1. mRNA levels of Rab32, Rab38 and Rab29 in THP-1 cells. (A) THP-1 cells were transduced with lentivirus encoding either an shRNA targeting Rab32 (Rab32KD) or non-targeting shRNA sequences (CTR) and the transcript levels of Rab32 and Rab38 were determined by RT-qPCR. (B) THP-1 cells were transduced with lentivirus encoding shRNA sequences targeting Rab29 and the transcript levels were analysed by RT-qPCR. In both cases GADPH expression was used as reference.

Fig S3

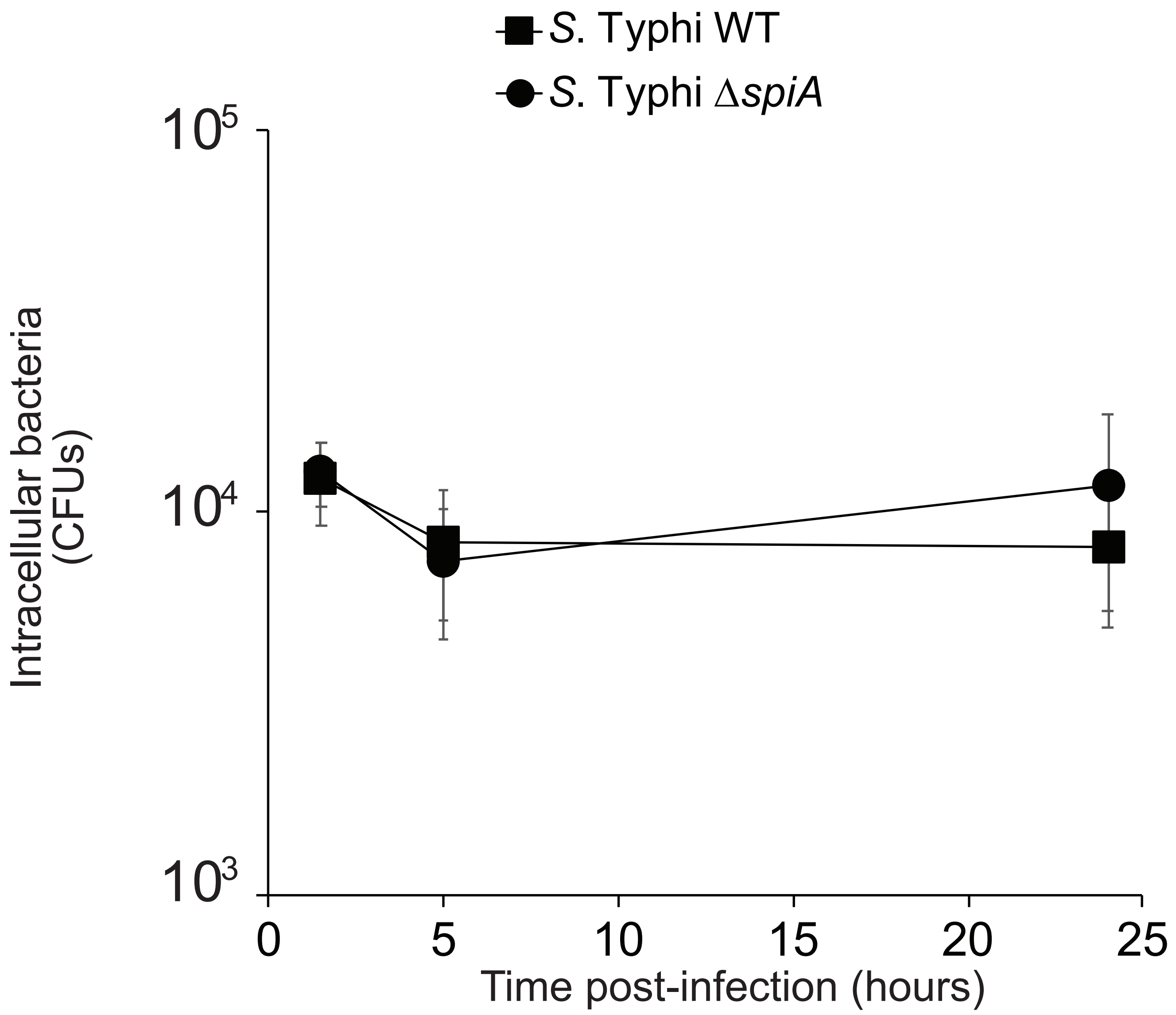

Fig S3. *S*. Typhi survival in macrophages does not depend on its SPI-2 type III secretion system. Human macrophages derived from induced pluripotent stem cells were infected with either wild-type *S*. Typhi or *S*. Typhi Δ*spiA*. Cells were lysed at the indicated time points to measure intracellular CFUs

Fig S4

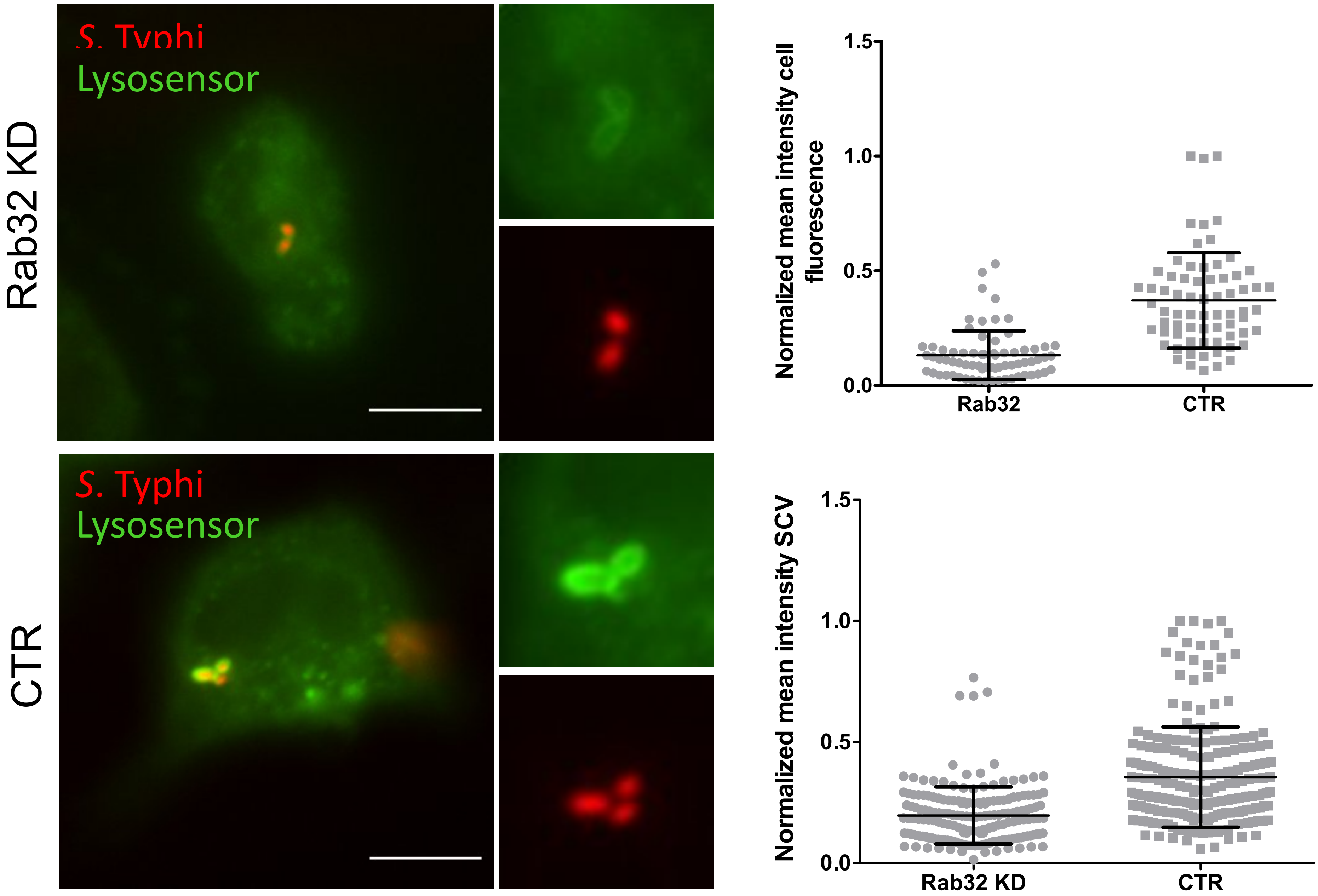

Figure S4. Depletion of Rab32 results in a decreased acidification of cellular compartments. (A) Differentiated THP-1 cells either depleted of Rab32 (Rab32 KD) or control (CTR) were infected with *S*. Typhi *glmS*::*Cm*::*mCherry* (multiplicity of infection= 10). Five hours post-infection, cells were stained with LysoSensor™ Green DND-189 to visualise acidic compartments (Scale bar=10 μm). The total fluorescence intensity of lysosensor Green in cells (B) or the *Salmonella*-containing vacuoles (SCVs) (C) was quantified using Image J. Circles and squares represent values from two independent experiments normalized based on the maximal intensity value of control (CTR) cells. For each population, the mean and standard deviation are shown. P-values were calculated using the Student´s t-test (unpaired, two-tails).

Table S1. List of *S*. Typhi strains used in this study

**Strain**	**Genotype**	**References**
ISP2825	wild type	Galán and Curtiss, 1991(17)
SB2522	*::gtgE*	Spanò and Galán, 2012 (8)
SB2174	Δ*invA*	Spanò *et al*., 2011 (16)
SB1958	Δ*spiA*	Spanò *et al*., 2011 (16)
SBB001	*glmS::Cm::mCherry*	this study
SBB002	::*gtgE glmS::Cm::mCherry*	this study

Table S2. List of plasmids used in this study

**Plasmid**	**Description**	**References**
pVSVG	p*VSV-G*	Spanò *et al*., 2011 (16)
pGag/Pol	pMLV-*Gag-Pol*	Spanò *et al*., 2011 (16)
